# *Pdel*, Encoding a Low-Affinity cAMP Phosphodiesterase, Regulates Conidiation and Pathogenesis in *Alternaria alternata* Tangerine Pathotype

**DOI:** 10.3389/fmicb.2020.597545

**Published:** 2020-12-07

**Authors:** Weiwei Lv, Xiangwen Kong, Changyong Zhou, Kezhi Tang

**Affiliations:** Citrus Research Institute, Southwest University, Chongqing, China

**Keywords:** *Alternaria alternata* tangerine pathotype, cAMP phosphodiesterase, conidiation, cell wall integrity, osmotic stress, pathogenicity

## Abstract

Based on intracellular second messenger cAMP, the cyclic AMP-protein kinase A (cAMP-PKA) pathway transforms extracellular stimuli to activate effectors and downstream signaling components, mediating physiological processes in filamentous fungi. The concentration of intracellular cAMP was regulated by adenylate cyclase biosynthesis and cAMP phosphodiesterase (PDEs) hydrolysis, which mediate signal transduction and termination. In this study, we used a gene deletion and complementary strategy to characterize the functions of *AaPdel* and *AaPdeh* genes, which encoded low-affinity PDEs (Pdel) and high-affinity PDEs (Pdeh), respectively, in *Alternaria alternata. AaPdel*, but not *AaPdeh*, was found to be a key regulator in conidiation and pathogenesis in *A. alternata*. Δ*AaPdel* showed defects in conidiation, producing approximately 65% reduced conidiation and forming lowly pigmented aberrant structures. In response to osmotic stress, Δ*AaPdel* was more sensitive to non-ionic osmotic stress than ionic osmotic stress. Moreover, *AaPdel* deletion mutants had defects in vegetative growth and hyphal growth. Further analyses showed that the high chitin content of Δ*AaPdel* might account for the sensitivity to Congo red. Based on the attenuated pathogenicity and lowly pigmented aberrant structures, the laccase activity analysis found that both *AaPdel* and *AaPdeh* were involved in laccase activity regulation. Our data further support the PKA-mediated cAMP signaling pathway, as we have found that *AaPdel* was involved in intracellular cAMP levels in *A. alternata.*

## Introduction

Seven pathotypes of *Alternaria alternata* have been identified in tangerine, Japanese pear, strawberry, apple, tomato, rough lemon, and tobacco (Hatta et al., 2002; Thomma, 2003). Alternaria brown spot disease (ABS) caused by the tangerine pathotype of *A. alternata* reduces quality and yield of tangerine production in China (Lee et al., 2015). Under the optimum condition of 28°C and high humidity, the *A. alternata* infected both leaves, twigs, and fruits, forming necrosis, and spread extensively (Solel and Kimchi, 1998). Infected leaves act as the primary infection source, and conidia are derived from the lesions of infected leaves (Wang et al., 2018). As a necrotrophic fungus, the tangerine pathotype of *A. alternata* produced the host selective toxin *A. citri* toxin (ACT) to kill and colonize the host cells (Tsuge et al., 2013; Ma et al., 2019). In the infection process, the ACT toxin spreads along the veins of the leaves, forming irregular “v”-shaped or like-round lesions. By causing electrolyte leakage, tangerine and its hybrids are susceptible to the ACT toxin (Otani et al., 1995; Ito et al., 2004).

In filamentous fungi, cells respond to external stimuli through cell-surface receptors and complex signal transduction pathways. The cyclic AMP-protein kinase A (cAMP-PKA) pathway is one of the major studied pathways mediating physiological processes in filamentous fungi (Zhu et al., 2017). Based on the cAMP, intracellular second messenger, extracellular stimuli could be transformed to activate effectors and stimulate downstream signaling components (Calvo et al., 2002). The cAMP-PKA pathway consists of G protein-coupled receptors (GPCRs), heterotrimeric G proteins, adenylate cyclase (AC), cAMP, and protein kinase A (PKA) (Houslay et al., 2007). The concentration of intracellular cAMP was regulated by adenylate cyclase biosynthesis and cAMP phosphodiesterase (PDEs) hydrolysis, which mediated signal transduction and termination (Jin et al., 1992). Two identified GTP-binding proteins, Ras1 and Ras2, regulate cAMP production by activating adenylate cyclase (Ramanujam and Naqvi, 2010). And PDEs were directly acting on active site, mediating cAMP hydrolysis to 5′-AMP (Conti and Beavo, 2007; Wilson et al., 2010). PDEs play an important role in the cAMP-PKA pathway, but their specific functions in *A. alternata* remain poorly understood.

In *Saccharomyces cerevisiae*, cAMP levels are modulated by two cAMP phosphodiesterase, low-affinity phosphodiesterase Pdel, and high-affinity phosphodiesterase Pde2. There is no obvious homology between Pde1 and Pde2. Simultaneously knockouting of *Pde1* and *Pde2* doubled the cAMP concentration, increased sensitivity to heat shock, and growth defects in non-fermentable carbon sources (Londesborough and Suoranta, 1983; Uno et al., 1983). *Pde2* regulates cAMP level induced by glucose stimulation, while there is no significant difference of cAMP level between the *Pde1* deletion mutant and the wild-type strain (Hu et al., 2010). *Pde1* does not regulate the cAMP level directly, but mediates a PKA negative feedback loop (Wera et al., 1997; Ma et al., 1999; Zhao et al., 2007; Caza and Kronstad, 2019).

In plant-microbe interactions, the fungus *Magnaporthe oryzae PdeH* (*Pde2*) has a dominant role in conidiation, pathogenicity, and intracellular cAMP level regulation. Compared with the wild-type strain, simultaneous knockout of *PdeH* and *PdeL* resulted in a 10-fold increase in cAMP level and completed disappearance of pathogenicity, whereas *PdeL* (*Pde1*) had no obvious function in *M. oryzae* (Zhang et al., 2011). The expression of GFP protein shows that PdeH localizes predominantly to the cytoplasm, while PdeL locates to the nucleus. *PdeH*-involved cAMP signaling is crucial for signal transduction and pathogenicity in *M. oryzae* (Ramanujam and Naqvi, 2010). In *Candida albicans*, the *PDE2* deletion mutant exhibits reduced invasion and virulence. In the simultaneous knockout of *CaPDE2* and *CaPDE1*, pathogenicity is abolished completely, indicating that *Pde1* contributes to virulence as a secondary role (Wilson et al., 2010). In phytopathogenic fungus *Botrytis cinerea*, the Δ*bcpde2* exhibits a significant reduction in vegetative growth, spore formation, germination, and pathogenicity, but the Δ*bcpde1* displays a similar phenotype to the wild-type strain (Harren et al., 2013). In *Fusarium graminearum*, inactivation of *PDE2* but not *PDE1* results in activating PKA activities and increases DON production (Jiang et al., 2016).

In *A. alternata*, the cAMP-PKA pathway remains poorly understood, and no regulated genes have been identified. In this study, characterization of PDEs in *A. alternata* have been conducted. We have identified high and low-affinity cAMP phosphodiesterase in *A. alternata* and through targeted gene deletion, analyzed the gene function in vegetative growth, conidiation, stress response, cell wall integrity (CWI), intracellular cAMP level, laccase activity, and pathogenicity.

## Materials and Methods

### Strains and Growth Condition

The *A. alternata* Z7 was used as a wild-type strain in this study (Wang et al., 2016). And the fungal strains defective of *AaPdel*, coding low-affinity cAMP phosphodiesterase, and *AaPdeh*, coding high-affinity cAMP phosphodiesterase, were used as deletion mutants (Δ*AaPdel* and Δ*AaPdeh*). For activating strains and determining characteristics of colonies, all strains were cultured on potato dextrose agar (PDA) plates for 4–8 days at 28°C. After targeted gene deletion, the resulting transformants were selected on TB3 medium (yeast extract 3 g, casamino acids 3 g, sucrose 200 g, and agar 7.5 g per liter of purified water) containing hygromycin (Kong et al., 2018). On the vegetative growth analyses, the wild type and deletion mutants were cultured on minimal medium (KCl 0.5 g, NaNO_3_ 2 g, KH_2_PO_4_ 1 g, MgSO_4_⋅7H_2_O 0.5 g, FeSO_4_ 0.01 g, sucrose 10 g, trace elements 200 μL and agar 20 g per liter of purified water) and complete medium (per liter of minimal medium supplied with yeast extract 1 g, casein hydrolysate 1 g, and peptone 2 g), respectively (Tang et al., 2020).

### Targeted Gene Deletion and Complementation Analysis

Gene replacement through the hygromycin-resistance cassette released from pCX62 was used to generate the deletion mutants (Dong et al., 2015). The upper fragment of the target gene (S1) and hygromycin B gene (H1) were amplified and fused together by two-step overlapping PCR. And the lower fragment of the target gene (S2) and hygromycin B gene (H2) were fused to another fragment. The two resulting fragments with a hygromycin-resistance cassette were introduced into the protoplasts of the wild type by the polyethylene glycol (PEG)-mediated knockout technique (Rehman et al., 2016). To confirm the correct gene replacement, the resulting transformants resistant to hygromycin (TB3 medium with 250 mg/ml hygromycin), were identified by PCR assay and southern blot analysis.

### Conidiation

The conidia of strains were harvested by blending with distilled water and scraping the colonies grown on V8 medium at 28°C for 8 days (Timmer et al., 1998). After filtering into two layers of sterile gauze, conidia were quantified by hemocytometer (QIUJING, China) and microscope (OLYMPUS, Japan). The conidia germinating rate and conidia diameter were assessed by microscopic examination of 200 conidia each for at least three repetitions. The statistical significance on conidiation of wild type, deletion mutants and complementary strains, was determined by one-way ANOVA and Duncan’s new multiple range test (*P* < 0.05).

### Nucleic Acid Manipulation

The homology of the targeted gene and protein sequences were searched in the Blast program and resources at NCBI^1^. Analyses of protein subcellular localization were predicted by WoLF PSORT^2^. Fungal genomic DNA and total RNA extraction were extracted from the mycelia grown in PDB medium for 36 h at 28°C. Fungal genomic DNA extraction was carried out using CTAB as previously described, and total RNA extraction was performed using Trizol (Invitrogen). Plasmid DNA was purified using the Plasmid DNA Miniprep Kit (Qiagen).

### Vegetable Growth and Stress Treatment

Four kinds of growth medium [PDA, V8, minimal medium (MM), and complete medium (CM)] were configured to determine the involvement of vegetative growth between deletion mutants and wild-type strain. To analyse the involvement of *AaPdel* and *AaPdeh* in cell wall integrity and osmotic stress response, 3 days activated strains were inoculated on PDA amended with different chemicals. The same concentration of osmotic-stress inducer, 1M sorbitol, 1M NaCl, 1M KCl, or 1M sucrose was added to the PDA plate, respectively. And SDS or Congo red (CR) inducing medium was configured to analyse the cell wall integrity of strains. The diameter of colonies were measured in two perpendicular directions after 5 days of incubation at 28°C, and each treatment was replicated for at least three times. And the growth rates were presented as the mean ± SD of at least three repeats. Statistical significance of growth rates of mutants and wild type was determined by one-way ANOVA and Duncan’s new multiple range test (MRT) (*P* < 0.05).

### Quantification of Intracellular cAMP

Mycelia, grown in PDB medium for 36 h at 28°C and freeze-dried for 1.5 h, were quickly ground in liquid nitrogen and mixed with 200 μL of 6% trichloroacetic acid (TCA). Intracellular cAMP extraction was in accordance with the method described previously (Liu et al., 2007), and cAMP levels were measured using the AlphaScreen Assay Kit (PerkinElmer, Waltham, MA) according to the supplier’s instruction.

### Pathogenicity Assay

In the pathogenicity assay, the 10 days conidia harvested from V8 medium were diluted to a concentration of 1.0×10^5^ conidia ml^–1^. At least 20 leaves of tangerine (*Citrus reticulata* Blanco) with nearly identified size and maturity, were inoculated with 20 μL conidial suspension of wild type, deletion mutants, and complementary mutants (Solel and Kimchi, 1998). Distilled water was inoculated on the leaves as a positive control. The phenotype was recorded at 3–5 dpi (days post inoculation), with three repetitions in each treatment. According to Perina et al. (2019) the average scale levels based on the percentage of lesion area were quantified.

### Laccase Activity Assay

Laccase activity was quantified as described previously (Mtibaa et al., 2018). Based on the oxidation of ABTS, the activated strains of wild type, Δ*AaPdel*, Δ*AaPdeh*, and complementary mutants were inoculated on the ABTS medium at 28°C. The morphology of these colonies was observed and photographed at 6 dpi. For the enzyme solution preparation, mycelial plugs of activated strains were cultured in 50 mL PDB liquid medium for 36 h. After filtration, mycelial pellets were harvested and mixed with 1 mL Tris-HCl. And the supernatant collected by centrifuge (10,000 rpm, 10 min) was the enzyme solution. Laccase activity was determined by 0.5 mM ABTS oxidation mixing in 0.1 M NaAc-HAc buffer, pH 4.0 at 28°C and measured the spectrophotometer absorbance at 420 nm. The resulted laccase activity was defined as the units U mL^–1^, with 1 U being the amount of enzyme oxidizing 1 μmol of ABTS per min (Irfan et al., 2018).

### Statistical Analysis

Data was presented as the mean ± SD of at least three independent experiments. Statistical significance was analyzed by one-way ANOVA and Duncan’s new multiple range test (MRT). Different letters represent significant differences at *P* < 0.05.

## Results

### *AaPdel*, but Not *AaPdeh*, Is Involved in Mycelial Growth and Hyphal Growth

**FIGURE 1:**
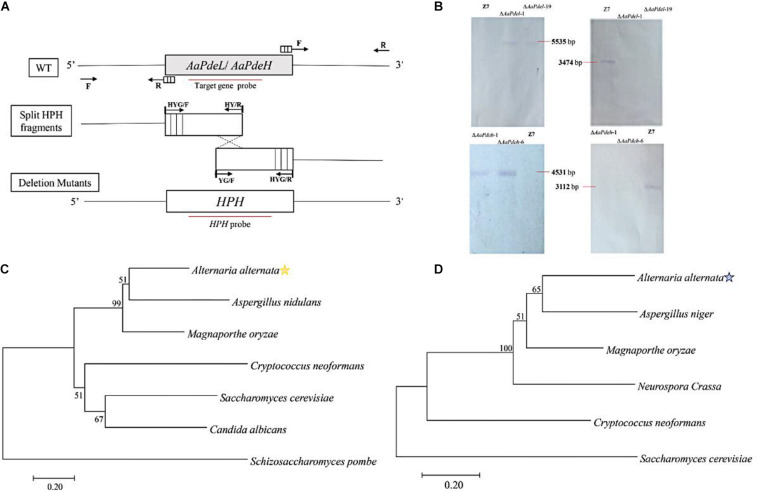
Constructionprotect and verification of the *AaPdel* and *AaPdeh* deletion and complementary mutants of *Alternaria alternata*. **(A)** Schematic of gene knockout. Targeted disruption of *AaPdel* and *AaPdeh* in *A. alternata* using split-marker gene replacement strategy. Through a homologous integration, overlapping DNA fragments of hygromycin-resistance gene cassette (*HPH*) were used for gene replacement. Arrows represented to primer binding sites, and the red line delineates the probe used for Southern blot analysis. **(B)** Southern blot analysis for the wild-type strain and deletion mutants. **(C)** The phylogenetic tree constructed based on the neighbor-joining method with 1,000 bootstrap replicates by MEGA7.0 program. Figures represent the occurrence percentage, and the value less than 50 was not shown. Phylogenetic tree generated based on the amino acid of AaPdel homologous. **(D)** Phylogenetic tree generated based on the amino acid of AaPdeh homologous.

Through blasting *S. cerevisiae* Pde1 and Pde2 in the proteome of *A. alternata*, the corresponding OWY42401.1 and OWY42000.1 were identified by high protein homology, and named AaPdel and AaPdeh, respectively. The sequence alignment demonstrated that the AaPdel protein exhibited 18.50–52.59% similarity with *S. cerevisiae* ScPde1, *M. oryzae* MoPdel, *C. albicans* CaPdel, and the AaPdeh protein exhibited 30.26–69.59% similarity with *S. cerevisiae* ScPde2, *M. oryzae* MoPdeh, *C. albicans* CaPde2, and *Botrytis cinerea* Bcpde2. The phylogenetic tree of two identified proteins were shown in Figures 1C,D, respectively. The identified *AaPdel* gene encodes a 1,100-amino-acid polypeptide, and the identified *AaPdeh* gene encodes a 961-amino-acid polypeptide. As demonstrated in Figure 1A, the split-marker approach was used to generate deletion mutants. To confirm the correct gene replacement, the resulting transformants were selected based on the resistance to hygromycin (TB3 medium containing 250 mg/ml hygromycin). And *AaPdel* transformants were verified by three primer pairs of PLP1F/H855R, H856F/PLP2R, and PLF/PLR. *AaPdeh* transformants were identified through PHP1F/H855R, H856F/PHP2R, and PHF/PHR (Supplementary Figure 1). Southern blot analysis was used for further analysis of the deletion mutants. Genomic DNA of *AaPdel* and *AaPdeh* transformants were respectively, digested with *Hin*dIII, *BgI* II, *Xho*I, and *Sal*I. A 5,535-bp fragment in the deletion mutants of *AaPdel* and a 4,531-bp fragment in the deletion mutants of *AaPdeh* were probed with *HPH* gene. And when probed with homologous target genes, respectively, 3,474 and 3,112-bp fragments were only found in the wild type, indicating correct replacement of the target genes (Figure 1B).

**FIGURE 2:**
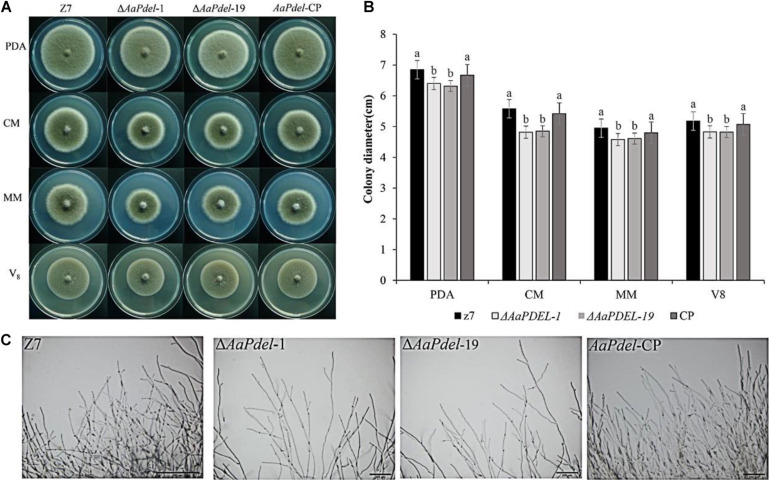
The deletion of *AaPdel* had significant effects on vegetative growth. **(A)** The phenotype of the wild-type strain, Z7; the deletion mutants, Δ*AaPdel-*1 and Δ*AaPdel-*19; and the complemented strain of Δ*AaPdel*, *AaPdel-*CP. All strains were inoculated on potato dextrose agar (PDA), minimal medium (MM), complete medium (CM), and V8 medium for 5 days at 28°C. **(B)** Statistical analyses of colony diameter. Colony diameter was presented as the mean ± SD of at least three independent experiments. Different letters on a graph denoted statistical significance at *P* = 0.05. **(C)** Hyphal morphology of wild type (Z7), *AaPdel* deletion mutants (Δ*AaPdel*-1 and Δ*AaPdel*-19) and complemented (*AaPdel*-CP) strains.

The wild type, z7; deletion mutants, Δ*AaPdel*-1, Δ*AaPdel*-19, Δ*AaPdeh*-1, and Δ*AaPdeh*-6; and complement strains, *AaPdel-cp* and *AaPdeh-cp*, were inoculated on four vegetative mediums of PDA, V8, CM, and MM, respectively. The colony morphology and diameter were assessed after growing for 6 days and measured in two orthogonal directions. The Δ*AaPdel* displayed similar vegetative growth defects on the PDA, MM, CM, and V8 media (Figure 2A). On the CM medium with sufficient nutrient elements, the growth rates of Δ*AaPdel* were decreased by 14%, significantly lower than the corresponding WT. On the MM covering the basic nutrient elements of fungal growth, the percentage growth rates of Δ*AaPdel* were decreased by 7.5%. The Δ*AaPdel* reduced growth on regular PDA medium by 6.6–7.8% compared with the wild type (Figure 2B). By light microscope observation, the wild type exhibited abundant and thick hyphal branch, while the hyphal growth of *AaPdel* deletion mutants was sparse (Figure 2C). However, the fungal strains defective of *AaPdeh*, coding high-affinity cAMP phosphodiesterase, exhibited no obvious growth defects in radical growth and colony morphology, which were in line with the wild type. The above results indicate that the *AaPdel* gene is involved in the mycelial growth of *A. alternata* while *AaPdeh* has a limited contribution.

### *AaPdel* Contributes to Conidia Formation and Morphology

**FIGURE 3:**
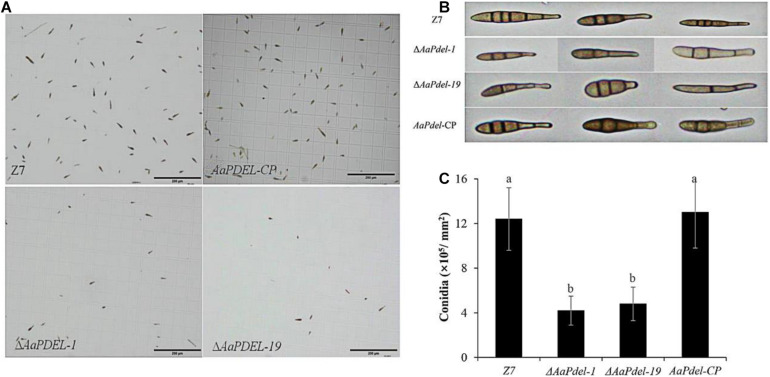
The Δ*AaPdel* of *Alternaria alternata* exhibited a significant reduction in conidia formation. **(A)** Light microscopy images of conidial suspension produced on V8 medium. **(B)** Conidia morphology of *A. alternata* strains examined by light microscopy. Δ*AaPdel* formed lowly pigmented aberrant structures. **(C)** Statistical analyses of conidia production. Statistical significance was analyzed by one-way ANOVA and Duncan’s new multiple range test (MRT). Different letters represent significant differences at *P* < 0.05.

To analyze potential gene functions in conidiation, the conidial suspension of wild type, deletion mutants, and supplementary strains were counted on a hemocytometer and photographed under a microscope at 40 times magnification. On the V8 medium, the Δ*AaPdel* formed colony with 10% reduced-growth-rate and produced approximately 65% reduced conidiation compared to the wild type (Figure 3A). The sporulation of the wild type Z7 was about 12.38 × 10^5^, while the sporulation of the *AaPdel* mutant was about 4 × 10^5^ (Figure 3C). Over 200 spore diameters of each strain were measured, and the spore morphology was observed under a microscope. The conidia of Δ*AaPdel* had varied morphologies, forming lowly pigmented aberrant structures. The conidia of Δ*AaPdel* were light gray, while the wild type and the complementary strain produced typical dark brown conidia (Figure 3B). However, through statistical analysis, *AaPdel* was not involved in conidia diameter and germination rate. There was no significant difference between Δ*AaPdeh* and wild type in terms of conidia production, germination rate, and morphology (Supplementary Figure 2). In summary, the *AaPdel* gene is involved in regulating the conidia production and pigment synthesis of conidia in *A. alternata.*

### *AaPdel* Plays a Role in Intracellular cAMP Level Regulation

**FIGURE 4:**
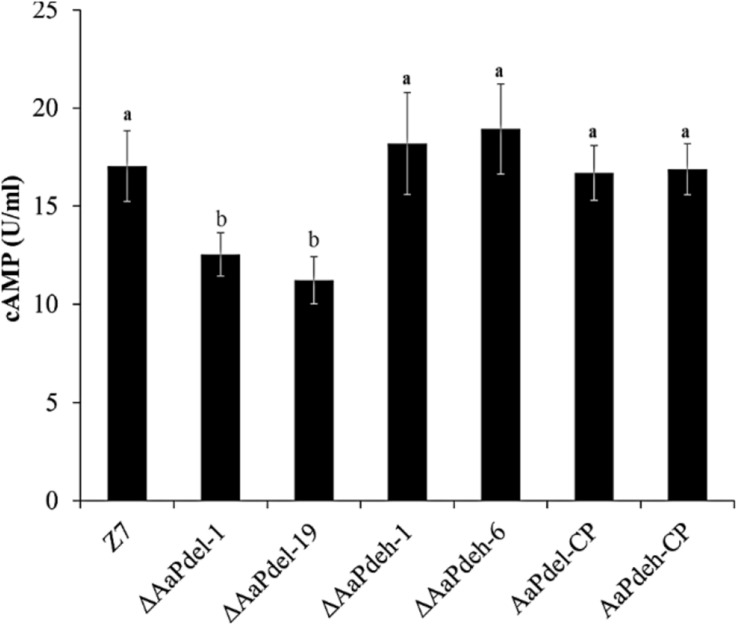
Intracellular cAMP quantification of Δ*AaPdel* and Δ*AaPdeh* during the hyphal stage in *Alternaria alternata*. Bar graphs depicted the levels of intracellular cAMP in the mycelia of Δ*AaPdeh*, Δ*AaPdel*, wild type, and complemented strain, which were cultured in liquid PDB for 48 h. Bars represented standard error. Different letters mark significant differences at *P* < 0.05.

Two cAMP phosphodiesterase-encoding genes were analyzed in the regulation of the intracellular cAMP level. Compared with the wild type, Δ*AaPdel* led to decreased accumulation of cAMP level, while the loss of *AaPdeh* led to the similar accumulation of cAMP level with the wild type. The trend was further confirmed by the complementation assay (Figure 4). The intracellular cAMP level of *AaPdel*-CP and *AaPdeh*-CP was consistent with the wild-type strain. *AaPdel* plays a role in intracellular cAMP level regulation.

### *AaPdel* Is Involved in Response to Osmotic Stress and Oxidative Stress

**FIGURE 5:**
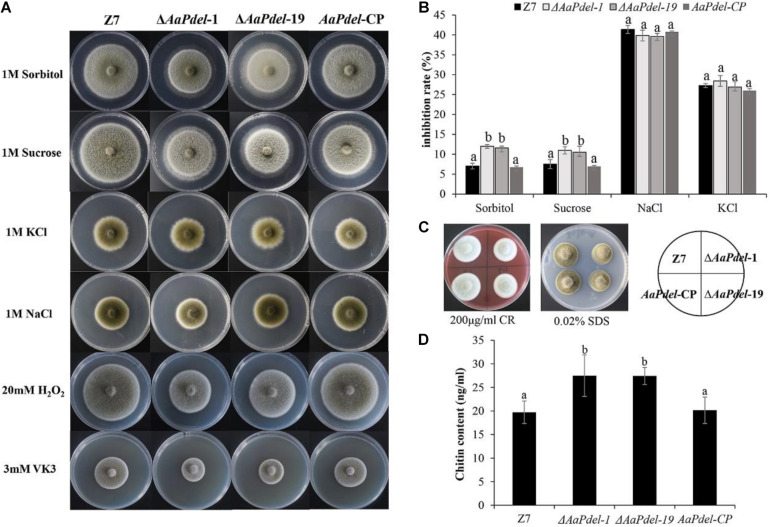
Effect of osmotic stress, oxidative stress and cell wall stress. **(A)** The phenotype of the wild-type strain (Z7); the deletion mutants (Δ*AaPdel*-1 *and*Δ*AaPdel*-19); and the complemented strain of Δ*AaPdel* (*AaPdel-*CP) inoculated on PDA medium amended with osmotic stress and oxidative stress. **(B)** Statistical analyses of inhibition rate on the osmotic stress. Results were presented as the mean ± SD of at least three independent experiments. Statistical significance was analyzed by one-way ANOVA and Duncan’s new multiple range test (MRT). Different letters mark significant differences at *P* < 0.05. **(C)** The phenotype of the strains inoculated on PDA medium with cell wall stress factors of sodium dodecyl sulfate (SDS) and Congo red (CR). **(D)** Quantitative analysis of cell wall chitin levels in wild-type strain, *AaPdel* deletion mutants, and complemented strains. Bars represented standard error. Different letters represent statistically significant differences (Duncan’s new multiple range test, *p* < 0.05).

To analyze the defects in response to the osmotic stress on the mutants, the activated strains were inoculated on PDA medium amended with 1 M sorbitol, 1 M NaCl, 1M KCl, or 1 M sucrose. Compared with the growth of the PDA medium with no stress factor present, the growth of all tested strains was restrained to the osmotic stress. The Δ*AaPdel*-1 and Δ*AaPdel*-19 mutants were hypersensitive to the osmotic stress inducers. The Δ*AaPdel* exhibited greater sensitivity to the non-ionic osmotic stress (sorbitol and sucrose) than the ionic osmotic stress (NaCl and KCl) (Figure 5A). Among these, the growth rate of Δ*AaPdel*-1 in sorbitol or sucrose were decreased by 10.58 and 10.11%, respectively, compared with the wild-type strain. Whereas with the ionic osmotic stress, the inhibitory effect of Δ*AaPdel*, Δ*AaPdeh* was similar compared with the wild type (Table 1). Online analysis of protein subcellular localization revealed that AaPdel protein was mainly located on the plasma membrane. According to the data, this different phenotype might be caused by the same concentrate Na^+^ and K^+^ treatment (Figure 5B).

**TABLE 1:** Inhibition rate of osmotic, oxidative stress and cell wall integrity assay in the wild type Z7, Δ*AaPdel*, *AaPdel*-CP in *Alternaria alternata*.

**Strain**	**Inhibition rate (%)**							
	**Sorbitol 1M**	**Sucrose 1M**	**NaCl 1M**	**KCl 1M**	**Congo Red 200 μg/ml**	**SDS 0.02%**	**H_2_O_2_ 20 mM**	**VK_3_ 3 mM**
Z7	7.030.71^A^	7.520.46^A^	41.360.32^A^	27.250.45^A^	45.753.37^A^	7.540.61^A^	12.653.17^A^	49.565.22^A^
Δ*AaPdel*-1	11.981.15^B^	10.980.92^B^	39.840.25^A^	28.431.51^A^	53.752.32^B^	15.631.25^B^	23.245.86^B^	66.677.29^B^
Δ*AaPdel*-19	11.610.98^B^	10.501.35^B^	39.580.25^A^	26.910.80^A^	54.494.51^B^	13.550.95^B^	21.243.57^B^	56.082.80^B^
*AaPdel*-CP	6.750.51^A^	6.950.35^A^	40.750.50^A^	26.001.35^A^	44.982.65^A^	7.500.51^A^	11.552.11^A^	52.263.80^A^

*Different letters represent statistically significant differences (Duncan’s new multiple range test, *p* < 0.05).*

In order to analyse the defects in response to the oxidative stress, the activated strains were inoculated on PDA medium added with oxidative stress factors (20 mM H_2_O_2_ or 3 mM VK_3_) after 5 days cultured at 28°C. The Δ*AaPdel* was hypersensitive to the oxidative stress inducers (Figure 5A). Δ*AaPdel*-1 had 23.24 and 66.67% inhibition rate, respectively, on PDA supplemented with H_2_O_2_ or VK_3_. The details of inhibition rate were presented in Table 1. However, under osmotic and oxidative stresses, the inhibition rates of Δ*AaPdeh-1* and Δ*AaPdeh-6* were similar to the wild type (Supplementary Figure 3).

### *AaPdel* Played a Role in the Maintenance of Cell Wall Integrity

Cell wall stress tolerance assays were performed by inoculating WT and mutant strains onto PDA medium supplemented with either 0.02% SDS or 100 μg/ml of CR. By serving PDA as control, the growth of strains was restrained to both SDS and CR. And the *AaPdel* mutants were hypersensitive to the cell wall stress reagents (Figure 5C). Compared with the wild type, the inhibition rate of the *AaPdel* mutants was significantly increased in 0.02% SDS (Table 1). Chitin, the major component of the cell wall in *A. alternata*, was further quantified in deletion mutants and the wild-type strain. The chitin content of Δ*AaPdel* was higher than that of the wild type and complemented strain, indicating *AaPdel* was involved in cell wall integrity and architecture (Figure 5D). However, the Δ*AaPdeh-1* and Δ*AaPdeh-6* were not more sensitive to the cell wall stress reagents. These stresses disturb cell wall biosynthesis in *A. alternata*, activating the CWI pathway. The CWI of the *AaPdel* mutant was affected, indicating that *AaPdel* is involved in the maintenance of CWI.

### *AaPdel* Is Required for Pathogenicity

**FIGURE 6:**
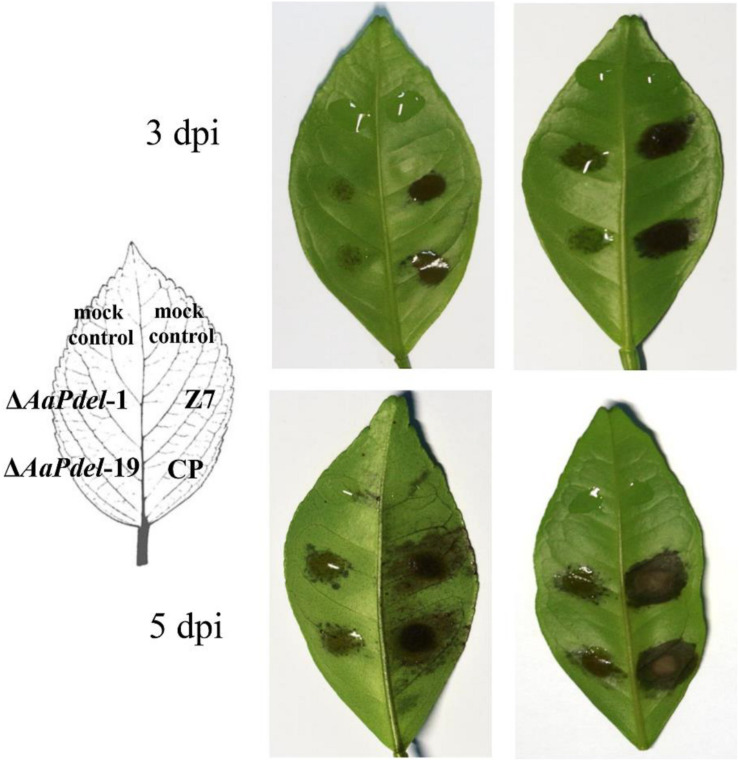
Mutation of *AaPdel* affected *Alternaria alternata* pathogenicity. Necrotic lesions of *A. alternata* on detached tangerine leaves induced by 20 μL conidial suspension (10^5^ conidia per mL) of wild type Z7, Δ*AaPdel* mutants, and the complemented strain, respectively. The mock controls were treated with distilled water only. Necrotic lesions were observed and photographed 3 and 5 dpi, respectively.

The pathogenicity was assessed by inoculating the leaves of tangerine with conidial suspension at a concentration of 1.0×10^5^ conidia ml^–1^. At 3 dpi, there were obvious lesions on the part of the leaves infected by wild type and complementary strains, but only mild lesions were observed on the leaves inoculated with Δ*AaPdel-1* and Δ*AaPdel*-19. At 5 dpi, Δ*AaPdel*-1 and Δ*AaPdel*-19 conidial suspensions incited small necrotic lesions in the leave (Figure 6). According to Perina et al. (2019), the average scale levels quantified the disease severity of deletion mutants and Z7 were 4 and 6, respectively. It showed nearly 75% reduced lesion area compared with WT. And *Pdel*-CP strain complemented the attenuated pathogenicity of Δ*AaPdel*. In contrast, the necrotic lesions induced by Δ*AaPdeh*-1 and Δ*AaPdeh*-6 were as serious as the wild type in pathogenicity (Supplementary Figure 4). Taken together, *AaPdel*, coding low-affinity cAMP phosphodiesterase, was involved in regulating the pathogenicity of *A. alternata*.

### *AaPdel* and *AaPdeh* Regulate Laccase Activity

**FIGURE 7:**
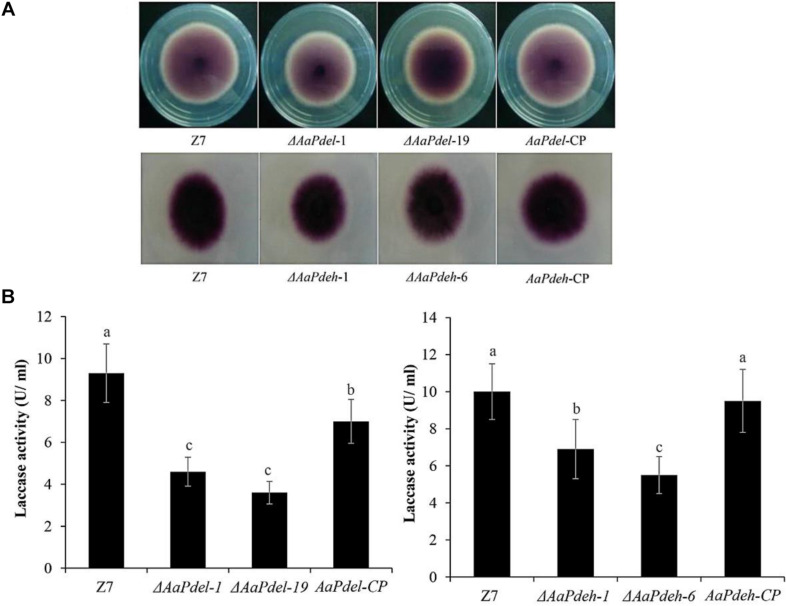
The laccases activity assays of Δ*AaPdel* and Δ*AaPdeh* of *Alternaria alternata*. **(A)** Laccase activity was tested on PDA medium with 0.03% ABTS culture at 28°C. Discoloration was observed and photographed 6 dpi. **(B)** Statistical analyses of laccase activity, which was determined by the ABTS oxidizing test. Statistical significance was analyzed by one-way ANOVA and Duncan’s new multiple range test (MRT). Different letters mark significant differences at *P* < 0.05.

Laccase is a copper-containing oxidase mainly involved in the catalysis of lignin degradation, pigment synthesis, and fungal pathogenicity. To determine the involvement of PDEs in laccase activity, we used a specific substrate, 2,2′-azino-di-3-ethylbenzthiazoline-6-sulfonate (ABTS). The oxidized dark purple stain can be observed at the colonies of both the Δ*AaPdel*,Δ*AaPdeh* mutants and the wild-type strain (Figure 7A). To further demonstrate the laccase activity difference between *AaPdel* and *AaPdeh* mutants, the crude enzyme solution was extracted and analyzed by spectrophotometer at 420 nm for 5 min. The statistical analyses showed that the laccase activity of both Δ*AaPdel* and Δ*AaPdeh* mutants were significantly lower than that of the wild type, and Δ*AaPdel* had a stronger inhibitory effect on laccase (Figure 7B). The above results indicate that both *AaPdel* and *AaPdeh* are involved in regulating laccase activity, and *AaPdel* plays a primary role in this pathway.

## Discussion

In fungi and yeast, cAMP signaling cascade regulate a large amount of cellular and biological processes. Since the discovery of cAMP-PKA pathway, PDEs have been characterized as important phosphodiesterase for cAMP level regulation in plant pathogens, including the budding yeast (*S. cerevisiae*) and filamentous fungi (*F. graminearum*, *M. oryzae*, *C. albicans*, and so on). Two PDEs, low-affinity phosphodiesterase Pdel (Pde1) and high-affinity phosphodiesterase Pdeh (Pde2), are involved in cAMP level regulation (Bahn et al., 2003). *Pdeh* is widespread in fungi and mammals, while *Pdel* is only found in fungi. In this study, the biological functions of the *AaPdel* and *AaPdeh* in *A. alternata* were preliminarily explored through gene replacement strategy, and it was found that *AaPdel* but not *AaPdeh* played a dominant role in biological processes of *A. alternata*, which differed from previously reported findings. Our data further support the PKA-mediated cAMP signaling pathway by showing that the regulator is involved in intracellular cAMP level.

In filamentous fungi, identified *PDEL* gene functions are mainly concentrated on conidiation, as in *F. graminearum* and *Setosphaeria turcica*. Our result was similar to those described above. The conidia production of the *AaPdel* deletion mutant was 65% lower than that of the wild type and complemented strain of Δ*AaPdel*. Also, the color of the Δ*AaPdel* conidia was relatively lighter. In *M. oryzae* and *Aspergillus flavus*, the *PdeH* deletion mutant also exhibited defects in vegetative growth and conidiation (Yang et al., 2017). In *S. turcica*, the deletion of the *StH-PDE* gene resulted in a loss of conidiation ability. Whereas the absence of *AaPdeh* did not result in the defects of conidia production, diameter, germination rate, and vegetative growth. The Δ*AaPdel* had growth defects on four vegetative growth media, and formed colonies with short, dense aerial hyphae. The growth defects of Δ*AaPdel* were extremely severe on the CM medium with sufficient nutrient elements. Comparing the utilization of basic nutrient elements, *AaPdel* might be involved in the utilization of certain nutrient elements. The *AaPdel* gene plays an important role in regulating conidiation and hypha growth.

The same concentration of osmotic stressors (sorbitol, sucrose, NaCl, and KCl) were used to analyze defects in stress response (Hanin et al., 2016). Δ*AaPdel* was more sensitive to non-ionic osmotic stress (sorbitol and sucrose) than ionic osmotic stress (NaCl and KCl). Among the analyzed osmotic stressors, sorbitol (a sugar alcohol), had the most significant inhibitory effect on the *AaPdel* deletion mutant. Whereas in the ionic osmotic stress, the inhibitory effect was the same when comparing the Δ*AaPdel*, Δ*AaPdeh*, and wild-type strain. According to the data, this phenotypic difference might be caused by the same concentration Na^+^ and K^+^ treatment. Analyses of protein subcellular localization revealed that the AaPdel protein mainly located on the plasma membrane. Hence, it can be hypothesized that *AaPdel* is involved in sodium-potassium pump regulation on the plasma membrane.

As the first barrier, the fungal cell wall and plasma membrane were of great significance to maintain the structural integrity and against external stresses. The fungal cell wall and plasma membrane are indispensable for maintaining the shape of the cell, and are also involved in signal transmission. In this study, Δ*AaPdel* was more sensitive to cell wall inhibitors CR and SDS, while Δ*AaPdeh* growth was similar to the wild type. SDS acts as a cell wall inhibitor by reducing the stability of the membrane to reveal the defect of the cell wall. CR inhibits binding to major components of the fungal cell wall, chitin and β-1,4-glucan (Gong et al., 2020). On the one hand, *AaPdel* appeared to be the sensor for signaling in response to the cell wall antagonist CR, which interferes with cell wall assembly by binding to chitin. Further analyses showed that the high chitin content of Δ*AaPdel* might account for the sensitivity to CR. However, the Δ*AaPdeh-*1 and Δ*AaPdeh-*6 were not more sensitive to cell wall stress reagents. Maintenance of cell wall integrity relies on the CWI pathway, a conserved cascade pathway, triggered by transmembrane sensors in response to extracellular stresses in fungus. These stresses disturb cell wall biosynthesis in *A. alternata*, activating the CWI pathway. The CWI of the *AaPdel* mutant was affected, indicating that *AaPdel* was involved in the maintenance of CWI.

Laccase is a copper-containing oxidase mainly involved in the catalysis of lignin degradation, pigment synthesis, and fungal pathogenicity (Mtibaa et al., 2018). The phenotype of lighter conidia color and attenuated pathogenicity led us to analyze the laccase activity. To determine the involvement of PDEs, the laccase activity of the Δ*AaPdel*, Δ*AaPdeh*, wild type, and complemented strain were tested and compared. We found that the wild type and complemented strain produced laccase, forming a dark purple hole on the ABTS medium, whereas the laccase activity of both Δ*AaPdel* and Δ*AaPdeh* was significantly lower than that of wild type and complemented strain. Consistent with our results, previous studies have demonstrated that the *MoPdeh* and *MoPdel* genes regulate intracellular laccase activity, and *MoPdel* plays a regulatory role in *M. oryzae*.

Research addressing gene functions in fungi has mainly focused on pathogens and those genes involved in pathogenicity or virulence. Among the studied PDEs, some genes encoding high-affinity phosphodiesterase affect pathogenicity. In *S. turcica*, the *StH-PDE* gene regulates secondary metabolism, melanin synthesis, and pathogenicity. The gene encoding high-affinity phosphodiesterase in *T. serrata* is involved in the regulation of mycelial growth and pathogenicity. *MoPdeH* (*Pde2*) has a dominant role in conidiation, pathogenicity, and intracellular cAMP level regulation, whereas *MoPdel* (Pde1) had no obvious function in *M. oryzae.* In this research, the finding was contrary to previous studies. In *A. alternata*, the Δ*AaPdel* mutant induced much smaller lesions than that of the wild type, whereas the Δ*AaPdeh* mutant induced lesions as drastic as those in the wild-type strain. Chitin is one of the fungal PAMPs, and the high content in Δ*AaPdel* may triggered rapid and strong plant parttern-triggered immunity. Above all, the attenuated pathogenicity of Δ*AaPdel* was probably caused by defects in the utilization of certain nutrient elements, osmotic stress response, plasma membrane, low laccase activity, high chitin content in the cell wall, and PKA-mediated cAMP signaling pathway.

PDEs target specific intracellular sites or signal transduction complexes and localize not only in the cytoplasm, but also in membrane, nucleus, and cytoskeleton locations (Houslay et al., 1998). Hu et al. (2010) reported that, like its low-affinity counterpart, the high-affinity phosphodiesterase may also play an important role in PKA negative feedback loop through the Pde2 localization assays. The localization of Pde2-GFP was affected by the carbon sources available and the cAMP-dependent PKA in the yeast *S. cerevisiae*. Pde2 is concentrated in the nucleus of cells grown on glucose, while it is distributed in the nucleus and cytoplasm of cells grown on some non-fermentable carbon sources. And PKA positively regulates the nuclear accumulation of Pde2. Elevated PKA activity increased the nuclear concentration of Pde2. Whereas in PKA attenuated strains, Pde2 is unable to concentrate in the nucleus of cells grown on glucose. Ramanujam and Naqvi (2010) reported the location of PdeH-GFP and PdeL-GFP during asexual and pathogenic development in *M. oryzae*. Although PdeH-GFP was cytoplasmic, it was dynamically associated with the plasma membrane and vesicle compartment, and the PdeL-GFP was predominantly localized in the nucleus. In *Aspergillus flavus*, PdeL-GFP shows a strong fluorescent signal in the nucleus of the hyphae, and PdeH-GFP is likely to be localized to punctate structures in the cytosol, rather than the nucleus. The localization of PDEs is complicated and dynamic. And limited to the resolution of the microscope, the reported location of the PDEs haven’t been directly localized to the organelle. In this study, we use online software to analyze the gene sequence. Online analysis of protein subcellular localization revealed that the AaPdel protein was predominantly located on the plasma membrane and the AaPdeh protein was located in the nucleus. From the perspective of protein subcellular localization, the AaPdeh protein was parallel to MoPdeL and AfPdeL, both in the nucleus. While AaPdel protein was similar to MoPdeH and AfPdeH. Further analysis of the signal peptides and transit peptides in AaPdel protein, revealed that the AaPdel protein was localized to the secretory pathway. We also investigate whether the two cAMP phosphodiesterase-encoding genes directly contribute to the intracellular cAMP level. Compared with the wild type, Δ*AaPdel* led to decreased accumulation of cAMP level, while the loss of *AaPdeh* led to the similar accumulation of cAMP level with the wild type. *AaPdel* regulated the intracellular cAMP level. This inverse trend of gene function in *A. alternata* was in accordance with the protein location difference between many fungi. We speculate that although PDEs homology has been identified in many filamentous fungi, their functions are not consistent, and there was no clear association between the conserved domain and physiological function. The inconsistent gene function may due to different protein location. It indicated that PKA-mediated cAMP signaling pathway was complicated in *A. alternata*, and needed further analysis.

In this study, the *AaPdel* and *AaPdeh* genes were identified to encode low-affinity PDEs (Pdel) and high-affinity PDEs (Pdeh) in *A*. *alternata*. We used a gene deletion and complementation strategy to characterize the gene function of *AaPdel* and *AaPdeh.* In *A. alternata*, *AaPdel* regulated intracellular cAMP levels during the hyphal stage. Deletion of *AaPdel*, but not *AaPdeh*, was found to play a role in conidiation and pathogenicity in *A. alternata*. Δ*AaPdel* showed a defect in conidiation, producing approximately 65% reduced conidiation and forming lowly pigmented aberrant structures. Upon exposure to osmotic stressors, Δ*AaPdel* had growth defects in response to osmotic stress, which was more sensitive to non-ionic osmotic stress than ionic osmotic stress. This different phenotype might be caused by the same concentrate Na^+^ and K^+^ treatment. Moreover, *AaPdel* deletion mutants had defects in vegetative growth, hyphal growth and pathogencity. Further analyses showed that the high chitin content of Δ*AaPdel* might account for the sensitivity to CR. Based on the attenuated pathogenicity and lowly pigmented aberrant structures, the laccase activity analyses found that both *AaPdel* and *AaPdeh* were involved in laccase activity regulation. Our data further support the PKA-mediated cAMP signaling pathway, as we have found the regulators of *AaPdel* is involved in intracellular cAMP levels in *A. alternata.* The inconsistent gene function may due to different protein location. In *A. alternata*, cAMP-PKA pathway played important roles in signal transduction, but the function of the genes involved in this pathway required further study.

## Data Availability Statement

The original contributions presented in the study are included in the article/Supplementary Material, further inquiries can be directed to the corresponding author/s.

## Author Contributions

KT, XK, and WL conceived and designed the experiments. CZ contributed to reagents, materials, and analysis tools. WL and XK performed the experiments. WL, KT, and CZ analyzed the data and wrote the manuscript. All authors read and approved the final manuscript.

## Conflict of Interest

The authors declare that the research was conducted in the absence of any commercial or financial relationships that could be construed as a potential conflict of interest.

## Footnotes

1 https://www.ncbi.nlm.nih.gov

2 https://www.genscript.com/psort/wolf_psort.html

## Supplementary Material

Supplementary Figure 1 PCR results of Δ*AaPdel* transformants were verified by three primer pairs of PLP1F/H855R, H856F/PLP2R, and PLF/PLR. *AaPdeh* transformants were identified through PHP1F/H855R, H856F/PHP2R, and PHF/PHR.

Supplementary Figure 2 The conidia of *AaPdeh* deletion mutants exhibit similar phenotype with wild-type strain. **(A)** The conidia morphology examined by light microscopy. **(B)** Statistical analysis of conidia production. The quantity of conidia was quantified after cultured on the V8 medium in dark for 8 days. Bars indicate standard errors of at least three repetitions.

Supplementary Figure 3 The sensitivity of the *AaPdeh* mutants to osmotic stress. Z7, Δ*AaPdeh*-1, Δ*AaPdeh*-6, and *AaPdeh*-CP strains were inoculated on PDA amended with osmotic stressors, NaCl, KCl, sorbitol or sucrose, respectively.

Supplementary Figure 4 Similar necrotic lesions induced by Δ*AaPdeh* strain and wild type Z7 on detached tangerine leaves. The conidial suspension at 1.0 × 105 conidia/ml of *Alternaria alternata* wild type Z7, Δ*AaPdeh* and the complemented strains were inoculated on leaves, respectively. The purified water was used as mock control.

## References

[B1] Bahn Y. S. Staab J. Sundstrom P. (2003). Increased high-affinity phosphodiesterase *PDE2* gene expression in germ tubes counteracts CAP1-dependent synthesis of cyclic AMP, limits hypha production and promotes virulence of *Candida albicans*. *Mol. Microbiol.* 50 391–409. 10.1046/j.1365-2958.2003.03692.x 14617167

[B2] Calvo A. M. Wilson R. A. Bok J. W. Keller N. P. (2002). Relationship between secondary metabolism and fungal development. *Microbiol. Mol. Biol. Rev.* 66 447–459. 10.1128/mmbr.66.3.447-459.2002 12208999 PMC120793

[B3] Caza M. Kronstad J. W. (2019). The cAMP/protein kinase a pathway regulates virulence and adaptation to host conditions in *Cryptococcus neoformans*. *Front. Cell. Infect. Microbiol.* 9:212. 10.3389/fcimb.2019.00212 31275865 PMC6592070

[B4] Conti M. Beavo J. (2007). Biochemistry and physiology of cyclic nucleotide phosphocliesterases: essential components in cyclic nucleotide signaling. *Annu. Rev. Biochem.* 76 481–511. 10.1146/annurev.biochem.76.060305.150444 17376027

[B5] Dong Y. H. Li Y. Zhao M. M. Jing M. F. Liu X. Y. Liu M. X. (2015). Global genome and transcriptome analyses of *Magnaporthe oryzae* epidemic isolate 98-06 uncover novel effectors and pathogenicity-related genes, revealing gene gain and lose dynamics in genome evolution. *PLoS Pathog.* 11:e1004801. 10.1371/journal.ppat.1004801 25837042 PMC4383609

[B6] Gong B.-Q. Wang F.-Z. Li J.-F. (2020). Hide-and-seek: chitin-triggered plant immunity and fungal counterstrategies. *Trends Plant Sci.* 25 805–816. 10.1016/j.tplants.2020.03.006 32673581

[B7] Hanin M. Ebel C. Ngom M. Laplaze L. Masmoudi K. (2016). New insights on plant salt tolerance mechanisms and their potential use for breeding. *Front. Plant Sci.* 7:1787. 10.3389/fpls.2016.01787 27965692 PMC5126725

[B8] Harren K. Brandhoff B. Knodler M. Tudzynski B. (2013). The high-Affinity phosphodiesterase *BcPde2* has impact on growth, differentiation and virulence of the phytopathogenic ascomycete *Botrytis cinerea*. *PLoS One* 8:e0078525. 10.1371/journal.pone.0078525 24265695 PMC3827054

[B9] Hatta R. Ito K. Hosaki Y. Tanaka T. Tanaka A. Yamamoto M. (2002). A conditionally dispensable chromosome controls host-specific pathogenicity in the fungal plant pathogen *Alternaria alternata*. *Genetics* 161 59–70. 12019223 10.1093/genetics/161.1.59 PMC1462115

[B10] Houslay M. D. Baillie G. S. Maurice D. H. (2007). cAMP-specific phosphodiesterase-4 enzymes in the cardiovascular system - A molecular toolbox for generating compartmentalized cAMP signaling. *Circ. Res.* 100 950–966. 10.1161/01.res.0000261934.56938.38 17431197

[B11] Houslay M. D. Sullivan M. Bolger G. B. (1998). The multienzyme PDE4 cyclic adenosine monophosphate-specific phosphodiesterase family: intracellular targeting, regulation, and selective inhibition by compounds exerting antiinflammatory and antidepressant actions. *Adv. Pharmacol.* 44 225–342. 10.1016/s1054-3589(08)60128-3 9547887

[B12] Hu Y. Liu E. K. Bai X. J. Zhang A. L. (2010). The localization and concentration of the PDE2-encoded high-affnity cAMP phosphodiesterase is regulated by cAMP-dependent protein kinase A in the yeast *Saccharomyces cerevisiae*. *FEMS Yeast Res.* 10 177–187. 10.1111/j.1567-1364.2009.00598.x 20059552

[B13] Irfan M. Mehmood S. Irshad M. Anwar Z. (2018). Optimized production, purification and molecular characterization of fungal laccase through *Alternaria alternata*. *Turk. J. Biochem. Turk. Biyokimya. Dergisi.* 43 613–622. 10.1515/tjb-2017-0239

[B14] Ito K. Tanaka T. Hatta R. Yamamoto M. Akimitsu K. Tsuge T. (2004). Dissection of the host range of the fungal plant pathogen *Alternaria alternata* by modification of secondary metabolism. *Mol. Microbiol.* 52 399–411. 10.1111/j.1365-2958.2004.04004.x 15066029

[B15] Jiang C. Zhang C. K. Wu C. L. Sun P. P. Hou R. Liu H. Q. (2016). TRI6 and TRI10 play different roles in the regulation of deoxynivalenol (DON) production by cAMP signalling in *Fusarium graminearum*. *Environ. Microbiol.* 18 3689–3701. 10.1111/1462-2920.13279 26940955

[B16] Jin S. L. C. Swinnen J. V. Conti M. (1992). Characterization of the structure of a low Km, rolipram-sensitive cAMP phosphodiesterase-mapping of the catalytic domain. *J. Biol. Chem.* 267 18929–18939. 1326538

[B17] Kong X. W. Tang F. Y. Lv W. W. Zhang Q. Tang K. Z. (2018). Optimization of protoplast preparation and regeneration conditions of *Alternaria alternata*. *J. Plant Prot.* 45 1431e1432.

[B18] Lee H. B. Patriarca A. Magan N. (2015). *Alternaria* in food: ecophysiology, mycotoxin production and toxicology. *Mycobiology* 43 93–106. 10.5941/myco.2015.43.2.93 26190916 PMC4505009

[B19] Liu H. Suresh A. Willard F. S. Siderovski D. P. Lu S. Naqvi N. I. (2007). *Rgs1* regulates multiple G alpha subunits in *Magnaporthe* pathogenesis, asexual growth and thigmotropism. *EMBO J.* 26 690–700. 10.1038/sj.emboj.7601536 17255942 PMC1794393

[B20] Londesborough J. Suoranta K. (1983). The zinc-containing high Km cyclic-nucleotide phosphodiesterase of bakers-yeast. *J. Biol. Chem.* 258 2966–2972. 6298214

[B21] Ma H. J. Zhang B. Gai Y. P. Sun X. P. Chung K. R. Li H. Y. (2019). Cell-wall-degrading enzymes required for virulence in the host selective toxin-producing necrotroph *Alternaria alternata* of citrus. *Front. Microbiol.* 10:2514. 10.3389/fmicb.2019.02514 31824437 PMC6883767

[B22] Ma P. S. Wera S. Van Dijck P. Thevelein J. M. (1999). The PDE1-encoded low-affinity phosphodiesterase in the yeast *Saccharomyces cerevisiae* has a specific function in controlling agonist-induced cAMP signaling. *Mol. Biol. Cell* 10 91–104. 10.1091/mbc.10.1.91 9880329 PMC25156

[B23] Mtibaa R. Barriuso J. de Eugenio L. Aranda E. Belbahri L. Nasri M. (2018). Purification and characterization of a fungal laccase from the ascomycete *Thielavia* sp. and its role in the decolorization of a recalcitrant dye. *Int. J. Biol. Macromol.* 120(Pt B), 1744–1751. 10.1016/j.ijbiomac.2018.09.175 30268749

[B24] Otani H. Kohmoto K. Kodama M. (1995). *Alternaria* toxins and their effects on host plants. *Can. J. Bot. Rev. Can. Bot.* 73 S453–S458. 10.1139/b95-282

[B25] Perina F. J. Belan L. L. Moreira S. I. Nery E. M. Alves E. Pozza E. A. (2019). Diagrammatic scale for assessment of alternaria brown spot severity on tangerine leaves. *J. Plant Pathol.* 101 981–990. 10.1007/s42161-019-00306-6

[B26] Ramanujam R. Naqvi N. I. (2010). PdeH, a high-affinity cAMP phosphodiesterase, is a key regulator of asexual and pathogenic differentiation in *Magnaporthe oryzae*. *PLoS Pathog.* 6:e1000897. 10.1371/journal.ppat.1000897 20463817 PMC2865543

[B27] Rehman L. Su X. F. Guo H. M. Qi X. L. Cheng H. M. (2016). Protoplast transformation as a potential platform for exploring gene function in *Verticillium dahliae*. *BMC Biotechnol.* 16:9. 10.1186/s12896-016-0287-4 27455996 PMC4960691

[B28] Solel Z. Kimchi M. (1998). Histopathology of infection of Minneola tangelo by *Alternaria alternata* pv. citri and the effect of host and environmental factors on lesion development. *J. Phytopathol.* 146 557–561. 10.1111/j.1439-0434.1998.tb04754.x

[B29] Tang K. Lv W. Zhang Q. Zhou C. (2020). Coding the alpha-subunit of SNF1 kinase, *Snf1* is required for the conidiogenesis and pathogenicity of the *Alternaria alternata* tangerine pathotype. *Fungal Biol.* 124 562–570. 10.1016/j.funbio.2020.02.008 32448447

[B30] Thomma B. (2003). *Alternaria* spp.: from general saprophyte to specific parasite. *Mol. Plant Pathol.* 4 225–236. 10.1046/j.1364-3703.2003.00173.x 20569383

[B31] Timmer L. W. Solel Z. Gottwald T. R. Ibanez A. M. Zitko S. E. (1998). Environmental factors affecting production, release, and field populations of conidia of *Alternaria alternata*, the cause of brown spot of citrus. *Phytopathology* 88 1218–1223. 10.1094/phyto.1998.88.11.1218 18944857

[B32] Tsuge T. Harimoto Y. Akimitsu K. Ohtani K. Kodama M. Akagi Y. (2013). Host-selective toxins produced by the plant pathogenic fungus *Alternaria alternata*. *FEMS Microbiol. Rev.* 37 44–66. 10.1111/j.1574-6976.2012.00350.x 22846083

[B33] Uno I. Matsumoto K. Ishikawa T. (1983). Characterization of a cyclic-nucleotide phosphodiesterase-deficient mutant in yeast. *J. Biol. Chem.* 258 3539–3542. 6300049

[B34] Wang M. Sun X. Yu D. Xu J. Chung K. Li H. (2016). Genomic and transcriptomic analyses of the tangerine pathotype of *Alternaria alternata* in response to oxidative stress. *Sci. Rep.* 6:32437. 10.1038/srep32437 27582273 PMC5007530

[B35] Wang M. Yang X. Ruan R. Fu H. Li H. (2018). *Csn5* is required for the conidiogenesis and pathogenesis of the *Alternaria alternata* tangerine pathotype. *Front. Microbiol.* 9:508. 10.3389/fmicb.2018.00508 29616013 PMC5870056

[B36] Wera S. Ma P. S. Thevelein J. M. (1997). Glucose exerts opposite effects on mRNA versus protein and activity levels of Pde1, the low-affinity cAMP phosphodiesterase from budding yeast, *Saccharomyces cerevisiae*. *FEBS Lett.* 420 147–150. 10.1016/s0014-5793(97)01508-1 9459299

[B37] Wilson D. Fiori A. Brucker K. D. Dijck P. V. Stateva L. (2010). *Candida albicans* Pde1p and Gpa2p comprise a regulatory module mediating agonist-induced cAMP signalling and environmental adaptation. *Fungal Genet. Biol.* 47 742–752. 10.1016/j.fgb.2010.06.006 20558315

[B38] Yang K. Liu Y. Liang L. Li Z. Qin Q. Nie X. (2017). The high-affinity phosphodiesterase PdeH regulates development and aflatoxin biosynthesis in *Aspergillus flavus*. *Fungal Genet. Biol.* 101 7–19. 10.1016/j.fgb.2017.02.004 28212851

[B39] Zhang H. Liu K. Zhang X. Tang W. Wang J. Guo M. (2011). Two phosphodiesterase genes, *PDEL* and *PDEH*, regulate development and pathogenicity by modulating intracellular cyclic AMP levels in *Magnaporthe oryzae*. *PLoS One* 6:e17241. 10.1371/journal.pone.0017241 21386978 PMC3046207

[B40] Zhao X. Mehrabi R. Xu J.-R. (2007). Mitogen-activated protein kinase pathways and fungal pathogenesis. *Eukaryot. Cell* 6 1701–1714. 10.1128/ec.00216-07 17715363 PMC2043402

[B41] Zhu W. J. Zhou M. Xiong Z. Y. Peng F. Wei W. (2017). The cAMP-PKA signaling pathway regulates pathogenicity, hyphal growth, appressorial formation, conidiation, and stress tolerance in *Colletotrichum higginsianu*. *Front. Microbiol.* 8:1416. 10.3389/fmicb.2017.01416 28791004 PMC5524780

