# Supplementary figures and images for: Pdel, Encoding a Low-Affinity cAMP Phosphodiesterase, Regulates Conidiation and Pathogenesis in Alternaria alternata Tangerine Pathotype
Source: Front Microbiol. 2020 Dec 7;11:597545. doi: 10.3389/fmicb.2020.597545 (PMC7750186; doi:10.3389/fmicb.2020.597545)

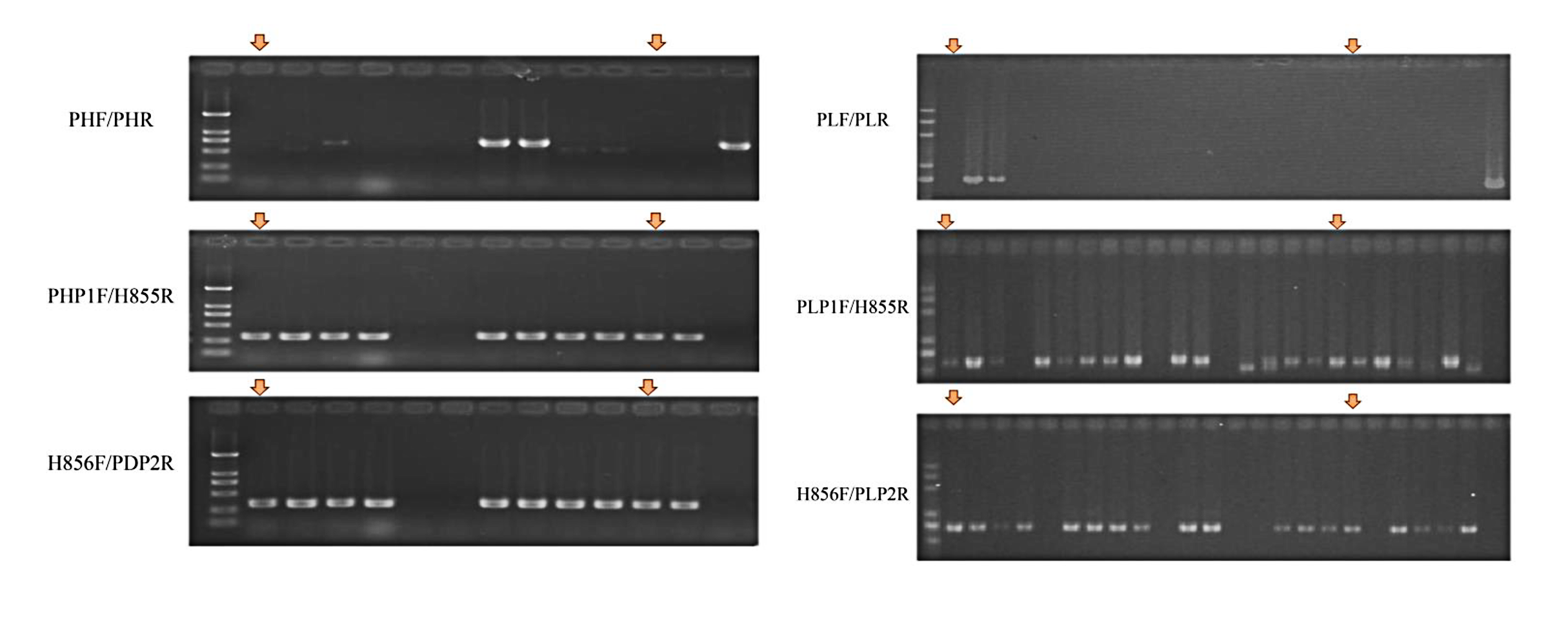

Supplement: Supplementary Figure 1 — PCR results of ΔAaPdel transformants were verified by three primer pairs of PLP1F/H855R, H856F/PLP2R, and PLF/PLR. AaPdeh transformants were identified through PHP1F/H855R, H856F/PHP2R, and PHF/PHR. [file Image_1.TIF]

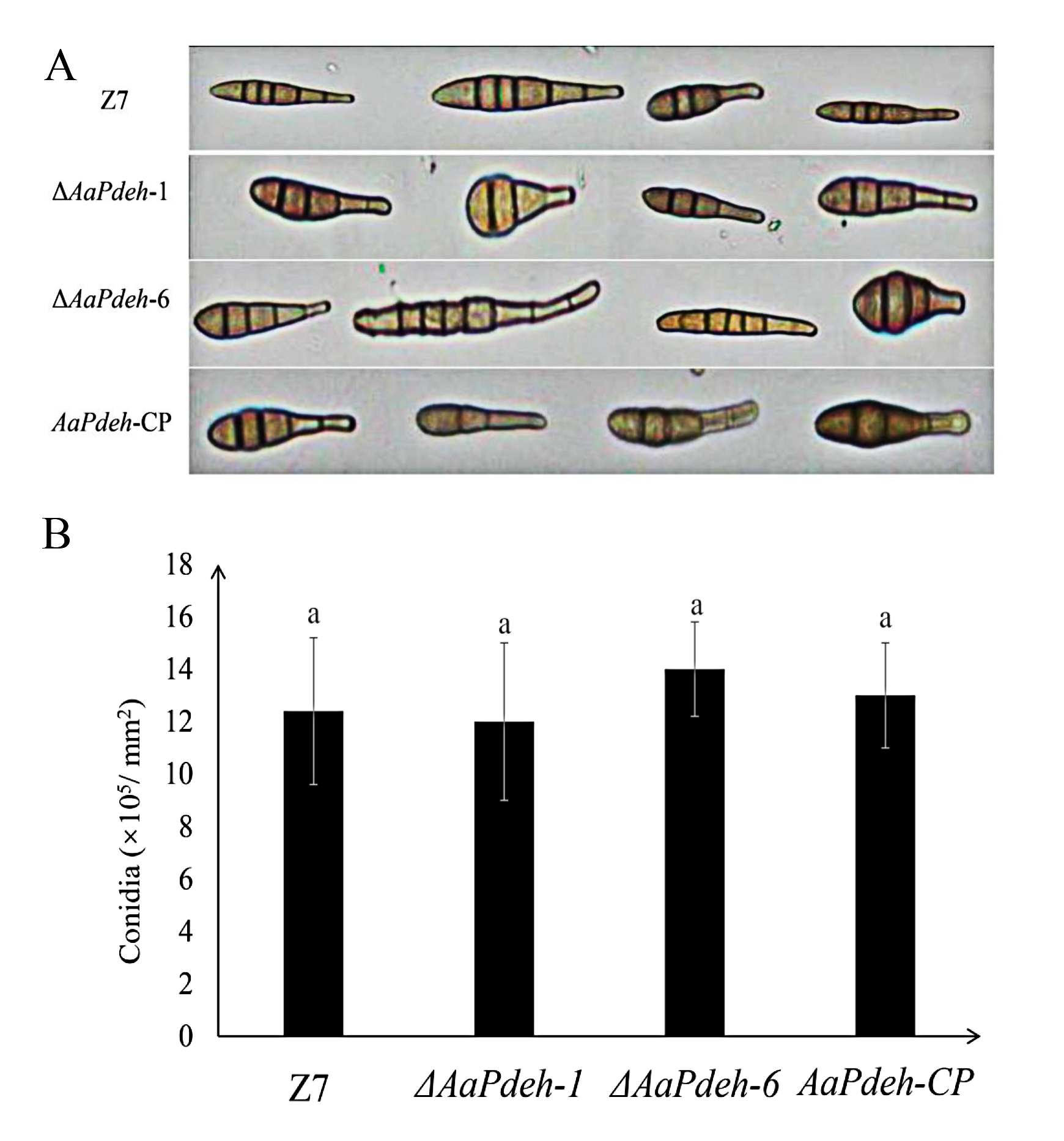

Supplement: Supplementary Figure 2 — The conidia of AaPdeh deletion mutants exhibit similar phenotype with wild-type strain. (A) The conidia morphology examined by light microscopy. (B) Statistical analysis of conidia production. The quantity of conidia was quantified after cultured on the V8 medium in dark for 8 days. Bars indicate standard errors of at least three repetitions. [file Image_2.TIF]

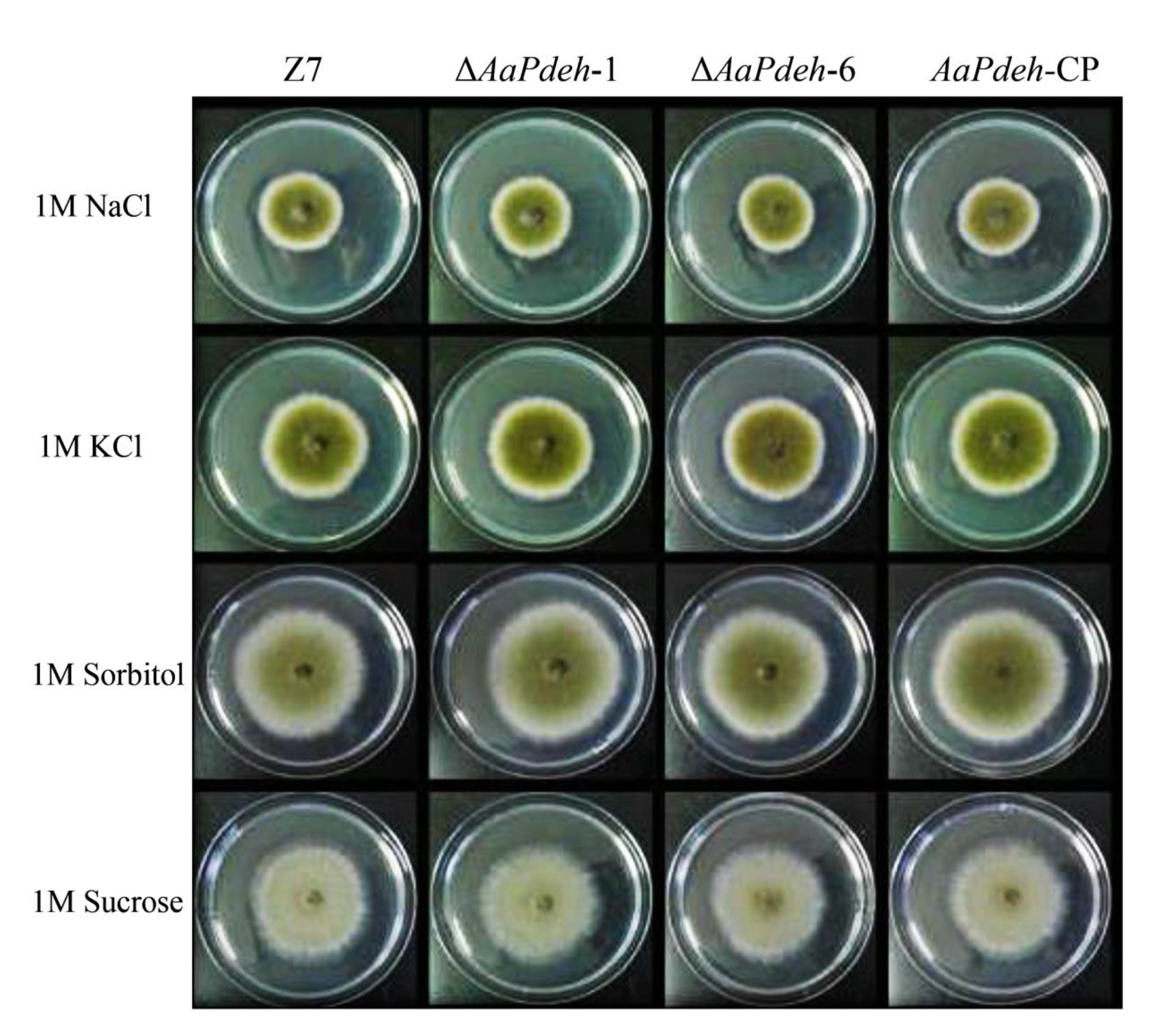

Supplement: Supplementary Figure 3 — The sensitivity of the AaPdeh mutants to osmotic stress. Z7, ΔAaPdeh-1, ΔAaPdeh-6, and AaPdeh-CP strains were inoculated on PDA amended with osmotic stressors, NaCl, KCl, sorbitol or sucrose, respectively. [file Image_3.TIF]

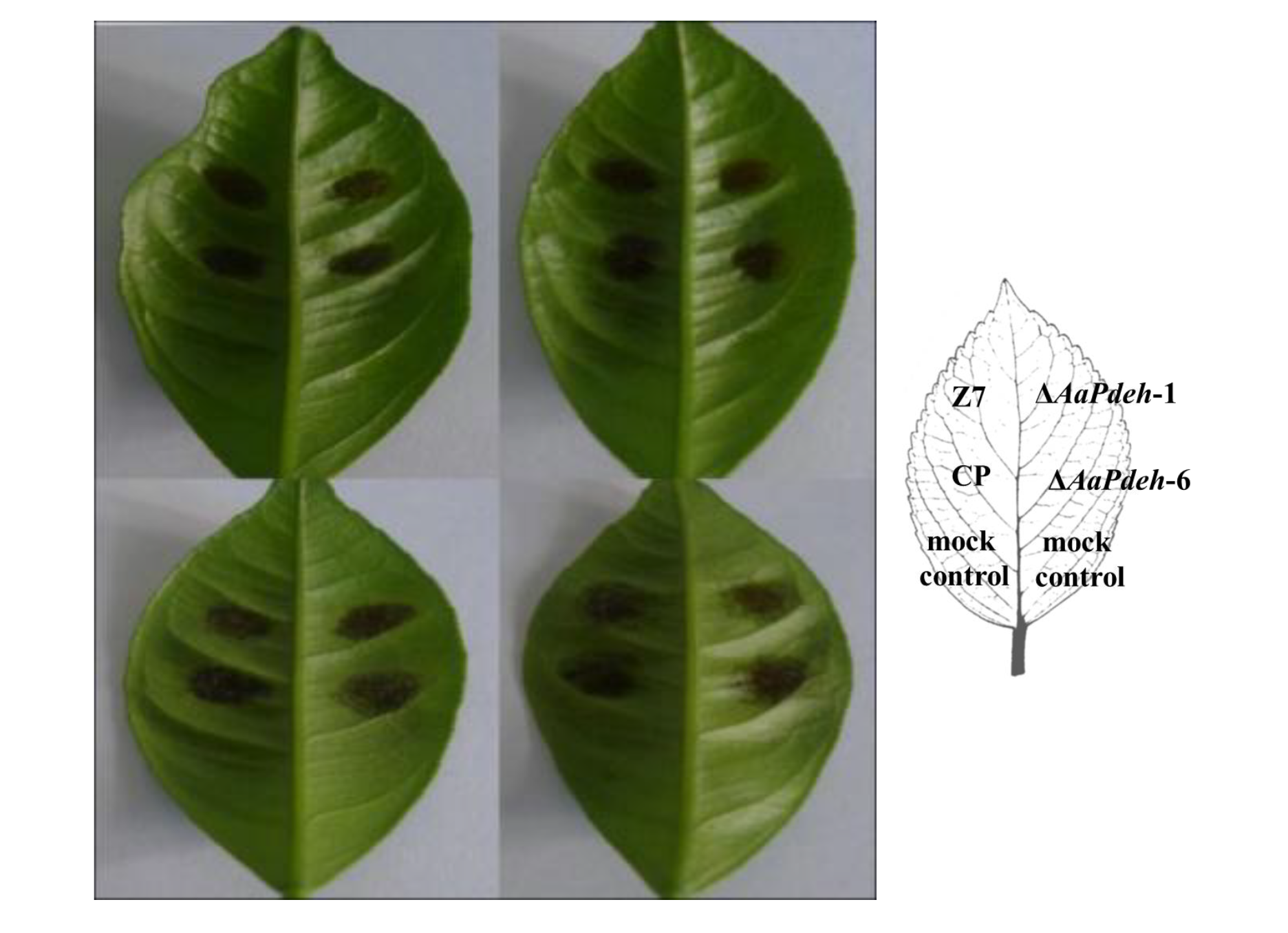

Supplement: Supplementary Figure 4 — Similar necrotic lesions induced by ΔAaPdeh strain and wild type Z7 on detached tangerine leaves. The conidial suspension at 1.0 × 10^5^ conidia/ml of Alternaria alternata wild type Z7, ΔAaPdeh and the complemented strains were inoculated on leaves, respectively. The purified water was used as mock control. [file Image_4.TIF]
